# The Correlation Between Biological Markers and Prognosis in Thyroid Cancer

**DOI:** 10.3390/biomedicines12122826

**Published:** 2024-12-12

**Authors:** Rami Hajjar, Ciprian Duta, Ionut Flaviu Faur, Catalin Prodan-Barbulescu, Hadi Alhajjar, Stelian Pantea

**Affiliations:** 1 IInd Surgery Clinic, Timisoara Emergency County Hospital, 300723 Timisoara, Romania; rami.hajjar@umft.ro (R.H.); flaviu.faur@umft.ro (I.F.F.); catalin.prodan-barbulescu@umft.ro (C.P.-B.); hadi.alhajjar@umft.ro (H.A.); 2 IInd Department of General Surgery, “Victor Babes” University of Medicine and Pharmacy, 300041 Timisoara, Romania; pantea.stelian@umft.ro; 3 Doctoral School, “Victor Babes” University of Medicine and Pharmacy Timisoara, Eftimie Murgu Square 2, 300041 Timisoara, Romania; 4 Department I—Discipline of Anatomy and Embryology, Faculty of Medicine, “Victor Babeş” University of Medicine and Pharmacy Timisoara, Eftimie Murgu Square 2, 300041 Timisoara, Romania

**Keywords:** thyroid cancer, *BRAF V600E* mutation, papillary thyroid carcinoma, thyroglobulin levels, calcitonin levels, biological biomarkers

## Abstract

**Background:** Thyroid cancer incidence is rising globally. Papillary thyroid carcinoma (PTC) is the most common subtype, usually with a favorable prognosis, while follicular, medullary, and anaplastic thyroid carcinomas carry higher risks. This study examines the relationship between biological markers—*BRAF V600E* mutation, thyroglobulin (Tg), and calcitonin—and thyroid cancer prognosis. **Materials and Methods:** This retrospective study included 395 thyroid cancer patients treated from 2010 to 2018 at the Emergency Clinical Hospital “Pius Brînzeu” in Timișoara. Patients were grouped by *BRAF V600E* mutation status (*n* = 178 with, *n* = 217 without). Preoperative Tg and calcitonin levels were measured, and survival rates were analyzed using Kaplan–Meier and Cox regression. **Results:** Patients with the *BRAF V600E* mutation were older, presented with advanced stages, and had higher Tg and calcitonin levels, correlating with tumor progression (e.g., Tg: 30.5 ng/mL in stage I vs. 62.0 ng/mL in stage IV). Lower biomarker levels (<30 ng/mL Tg, <15 pg/mL calcitonin) were associated with significantly better five-year survival rates (82.1% vs. 67.5%). Advanced stage, age, and elevated biomarkers independently predicted increased mortality risk. **Conclusions:** The *BRAF V600E* mutation is associated with more aggressive thyroid cancer and poorer outcomes. Tg and calcitonin are reliable prognostic markers, aiding in risk stratification and personalized treatment strategies to improve outcomes in thyroid cancer care.

## 1. Introduction

Thyroid cancer, while not the most prevalent cancer [1], has been on a concerning rise in incidence globally over the past few decades. This alarming trend, also seen in many endocrine malignancies, underscores the urgent need for immediate research and innovative solutions. This disease poses a unique challenge due to its varied clinical presentations and outcomes. The thyroid gland, responsible for regulating vital bodily functions through hormone production [2], becomes a battleground when malignant cells arise from its tissues, leading to thyroid cancer [3]. This includes several subtypes, each with distinct histological features and clinical behaviors.

Among the different types of thyroid cancer, papillary thyroid carcinoma (PTC) is the most prevalent, accounting for approximately 70–80% of cases [4]. This subtype is generally associated with a favorable prognosis and high survival rates [5]. However, other types, such as follicular thyroid carcinoma (FTC), medullary thyroid carcinoma (MTC), and the more aggressive anaplastic thyroid carcinoma (ATC), can present with more severe outcomes and lower survival rates [6]. The variability in prognosis across different thyroid cancer subtypes underscores the importance of identifying reliable prognostic markers, a crucial step in improving patient outcomes.

One critical challenge in managing thyroid cancer is accurately predicting disease progression and patient survival. Over the years, researchers and clinicians have identified several biological markers that can provide valuable prognostic information. Among these, the *BRAF V600E* mutation [7], thyroglobulin (Tg) [8], and calcitonin [9] levels have emerged as significant indicators. These biomarkers help predict the prognosis and guide treatment decisions, improving patient outcomes.

The *BRAF* mutation is a common genetic alteration observed in papillary thyroid carcinoma and plays a significant role in disease progression and survival rates [10]. This mutation activates the MAPK/ERK signaling pathway, promoting cancer cell growth and proliferation [11]. Multiple studies have demonstrated that the presence of the *BRAF* mutation is linked to more aggressive tumor behavior, higher recurrence rates, and poorer overall survival [12]. Understanding the impact of this mutation in thyroid cancer can help classify patients based on their risk and personalize their treatment, offering hope for improved patient outcomes.

Tg is another crucial biomarker in managing thyroid cancer. It is a glycoprotein produced exclusively by normal and malignant thyroid follicular cells. Elevated levels of thyroglobulin in the blood, especially after thyroidectomy, can indicate the presence of residual thyroid tissue or recurrent disease. Monitoring thyroglobulin levels is crucial in treating thyroid cancer patients, particularly those with differentiated thyroid cancers such as PTC and FTC [13].

Calcitonin, a hormone produced by the parafollicular C cells of the thyroid, is a critical marker for MTC. Elevated calcitonin levels in the blood indicate MTC and can aid in early diagnosis, disease progression monitoring, and recurrence detection. Given the aggressive nature of MTC, early detection and ongoing monitoring using calcitonin levels can significantly impact patient outcomes [14].

Biomarkers such as thyroglobulin (TG), calcitonin, and *BRAF V600E* mutations play distinct roles in diagnosing and prognosis thyroid carcinoma subtypes. While calcitonin is a well-established marker for medullary thyroid carcinoma (MTC), its measurement across all thyroid cancer subtypes, including papillary thyroid carcinoma (PTC) and follicular carcinoma (FTC), can provide critical insights. This comprehensive approach allows the identification of incidental or misclassified MTC cases and offers the potential to explore calcitonin’s prognostic value in other thyroid cancers. Similarly, TG and *BRAF V600E* mutation analyses help delineate subtype-specific prognostic implications, contributing to a broader understanding of thyroid cancer pathophysiology.

In this study, we aim to explore the correlation between these critical biological markers—*BRAF V600E* mutation, thyroglobulin, and calcitonin—and the prognosis of thyroid cancer. By analyzing a comprehensive cohort of 395 thyroid cancer patients, we seek to provide a deeper understanding of how these markers can influence disease progression and survival rates. Our cohort is divided into two groups based on the presence of the *BRAF V600E* mutation, allowing us to examine the differential impact of this genetic alteration on clinical outcomes.

Our findings will contribute to the growing body of evidence supporting the use of biological markers in clinical practice, offering a valuable resource for clinicians. Through this study, we aim to pave the way for future research that continues to refine and expand our understanding of the complex interplay between genetic, molecular, and clinical factors in thyroid cancer, instilling hope and optimism for further advancements in the field.

## 2. Materials and Methods

### 2.1. Study Design and Participants

This retrospective study was conducted on patients diagnosed with thyroid cancer and treated between 2010 and 2018 in the Endocrinology Department of the Emergency Clinical Hospital “Pius Brînzeu” in Timișoara. The study included patients whose presence or absence of the *BRAF V600E* mutation was determined and who benefited from a five-year follow-up to assess survival and prognosis.

A total of 395 patients with thyroid cancer were included, of whom 178 had the *BRAF V600E* mutation, and 217 did not have this mutation. Diagnosis and tumor stage were established according to current international guidelines. Demographic, clinicopathological data and biomarker values (thyroglobulin and calcitonin) were collected from the patient’s medical records.

We established criteria for including and excluding participants to optimize the study’s organization and clarity.

The inclusion criteria were as follows:

1. Patients diagnosed with thyroid cancer between 2010 and 2018.
2. Patients older than 18 years at the time of diagnosis.
3. Patients who have undergone surgery for thyroid cancer.
4. Patients with confirmed determination of the presence or absence of the *BRAF V600E* mutation.
5. Patients with complete medical record data on preoperative thyroglobulin and calcitonin values.
6. Patients who followed the standard treatment protocol for thyroid cancer.
7. Patients with a follow-up of at least five years from diagnosis.
8. Patients who agreed to participate in the study and signed the informed consent.
9. Patients treated and monitored in the Endocrinology Department of the Emergency Clinical Hospital “Pius Brînzeu” in Timișoara.
10. Patients with no history of other previously diagnosed cancers.

The exclusion criteria were as follows:

1. Patients with an incomplete or uncertain diagnosis of thyroid cancer.
2. Patients with active autoimmune diseases that could influence biomarker levels.
3. Patients who received experimental treatments for thyroid cancer in other clinical trials.
4. Patients with severe renal or hepatic impairment.
5. Patients with severe or chronic infections in the preoperative period.
6. Patients with severe allergies to drugs used in standard thyroid cancer treatment.
7. Patients with psychiatric or cognitive disorders that could affect their ability to follow the study protocol.
8. Patients who refused to provide access to their complete medical data.
9. Patients moving out of the geographic monitoring area can no longer be followed appropriately.
10. Patients with a history of non-compliance with prescribed medical treatments.

### 2.2. Evaluation of Biomarkers

Preoperative thyroglobulin and calcitonin levels were determined using standardized laboratory methods to ensure accurate and reproducible results. Venous blood samples were collected from each patient at the time of thyroid cancer diagnosis. The collection followed standardized sampling, handling, and processing procedures to minimize pre-analytical variations. For survival analysis, the thresholds were set at <30 ng/mL for thyroglobulin and <15 pg/mL for calcitonin, which are considered critical for assessing patients’ prognosis.

Histopathological diagnosis for cases with *BRAF V600E* mutations was conducted following standard protocols. For follicular thyroid carcinoma, encapsulated follicular variant of papillary thyroid carcinoma (FVPTC) was distinguished based on capsular and vascular invasion criteria.

### 2.3. Data Analysis

Data were analyzed using advanced statistical methods to assess the impact of clinicopathological variables on survival. Kaplan–Meier analysis was used to estimate five-year survival rates and median survival. Hazard ratio (HR) and 95% confidence intervals (CI) were calculated using proportional Cox regression models to assess risk factors associated with mortality.

The primary endpoints included age, tumor stage, and biomarker values. Correlation coefficients assessed correlations between variables, and statistical significance was determined by appropriate tests, such as the log-rank test for Kaplan–Meier analyses and Wald tests for Cox regression models. The *t*-test and z-test were used for comparisons, and all statistical analyses were performed using GraphPad Prism 6.

### 2.4. Ethical Consideration

The study ensured that all participants provided informed consent before participating, following ethical standards. Participants were fully informed about the study’s goals and procedures, and they agreed to contribute to the research, provide blood samples for scientific analysis, and allow the use of their personal and health-related data. This comprehensive consent process was necessary to respect participants’ autonomy and ensure that their rights and preferences were honored. Additionally, this study took strict measures to protect the confidentiality and privacy of all participants by carefully anonymizing all collected data to remove personal identifiers, demonstrating a solid commitment to maintaining high ethical research standards. Furthermore, the research received ethical approval from the ethics committee of the “Pius Brînzeu” Clinical Emergency Hospital in Timișoara, Romania documented under number 72 on 28 June 2021. This approval highlights the adherence to ethical research protocols and guidelines, showing a commitment to conducting research with integrity and respect for participant welfare.

## 3. Results

In this study, we assessed the demographic and clinical characteristics of the patients based on the presence or absence of the *BRAF* mutation, recorded as V600E. These characteristics are detailed in Table 1. The gender distribution revealed a slight imbalance, with a higher prevalence of women in both cohorts. The mean age exhibited a significant difference between the groups, suggesting a tendency for the *BRAF V600E* mutation to occur at an older age. Smoking status did not show significant differences between groups, indicating that smoking is not correlated with the presence of the *BRAF V600E* mutation. The family history of cancer was similar between groups, suggesting that family genetic factors have a comparable influence in both cases. The mean body mass index (BMI) was slightly higher in patients with the *BRAF V600E* mutation but without significant differences.

Alcohol consumption showed variation between groups but without statistical significance, suggesting that the frequency of alcohol consumption does not influence the occurrence of the mutation. Moderate physical activity was significantly more frequent in patients without the *BRAF V600E* mutation, indicating a possible protective effect of moderate physical exercise. Education level and marital status were comparable between groups, without significant differences, showing that these socio-economic variables are not associated with the presence of the mutation. Regarding occupational status, most patients in both groups were employed, and the proportion of unemployed or retired individuals was similar, indicating that occupational status does not appear to be influenced by the presence of the *BRAF V600E* mutation.

These data provide a comprehensive picture of the demographic and behavioral profile of the studied patients, highlighting specific trends and correlations that may be useful for identifying factors associated with the *BRAF V600E* mutation.

In this study, we analyzed the patients’ histological types and tumor stages in relation to the presence of the mutation. Our analysis of histological types revealed a significant predominance of papillary carcinoma in patients with the *BRAF* mutation compared to those without this mutation. Additionally, follicular and medullary types were considerably less common in the mutation group, suggesting a close association between the *BRAF* mutation and papillary histological type.

The evaluation of tumor stages (Table 2) showed notable differences between the two groups. Patients with the *BRAF* mutation tended to be diagnosed at more advanced stages, with a higher prevalence of stage IV. In contrast, patients without the mutation were more frequently diagnosed at early stages, especially stage I. The distribution of stages II and III did not show significant differences between the groups, suggesting that the mutation mainly influences the distribution between extreme stages (I and IV). These findings highlight the importance of the *BRAF* mutation in determining histological characteristics and tumor progression, with significant implications for patients’ diagnostic strategy and clinical management.

An analysis of preoperative biomarker levels revealed significant differences among histopathological subtypes in patients with and without the *BRAF V600E* mutation (Table 3). Patients with papillary thyroid carcinoma (PTC) and the *BRAF* mutation showed higher mean thyroglobulin levels (45.2 ± 22.5 ng/mL) compared to those without the mutation (28.5 ± 14.0 ng/mL), suggesting more intense tumor activity or a larger tumor mass. Calcitonin levels remained low in PTC patients regardless of mutation status, confirming the specificity of this biomarker for parafollicular-derived carcinomas.

For follicular thyroid carcinoma (FTC), cases positive for *BRAF V600E* had thyroglobulin levels similar to those observed in PTC (28.5 ± 14.0 ng/mL). However, upon reanalysis, these cases were reclassified as the encapsulated follicular variant of PTC (FVPTC). Calcitonin levels in FTC were characteristically low, with no significant differences between patients with or without the mutation.

In contrast, patients with medullary thyroid carcinoma (MTC) exhibited the highest calcitonin levels, reaching 85.4 ± 25.3 pg/mL in the *BRAF*-negative group and 18.7 ± 10.2 pg/mL in the *BRAF*-positive group. These values underscore the specificity of calcitonin as a tumor marker for MTC and reflect the greater biological aggressiveness of these cases.

These findings confirm the relevance of preoperative biomarkers in risk stratification and the adaptation of clinical management based on the histopathological subtype and *BRAF* mutation status.

The analysis of biomarker levels by tumor stage reveals a strong positive correlation between thyroglobulin and calcitonin levels and tumor progression across histopathological subtypes (Table 4). In papillary thyroid carcinoma (PTC), both thyroglobulin and calcitonin levels increase significantly with the advancing tumor stage, from stage I to stage IV (r = 0.76 and r = 0.65, respectively; *p* < 0.001). This trend suggests that these biomarkers effectively reflect disease progression in PTC.

In follicular thyroid carcinoma (FTC), a similar pattern is observed, with thyroglobulin and calcitonin levels showing moderate increases across tumor stages (r = 0.71 and r = 0.62, respectively; *p* < 0.001). Although the biomarker values in FTC are slightly lower than those in PTC, their progressive rise indicates their utility in monitoring tumor progression within this subtype.

For medullary thyroid carcinoma (MTC), calcitonin demonstrates the most significant elevation across tumor stages, with values reaching 110.5 ± 35.2 pg/mL at stage IV, reflecting its role as a primary biomarker for this subtype (r = 0.80; *p* < 0.001). Thyroglobulin levels in MTC, while lower than in PTC or FTC, also increase progressively with the advancing tumor stage (r = 0.68; *p* < 0.001).

These findings emphasize the value of both thyroglobulin and calcitonin in monitoring disease progression and stratifying patient risk across all histopathological subtypes. Regardless of the subtype, the correlation coefficients confirm the reliability of these biomarkers in guiding clinical management and informing therapeutic decisions, contributing to more personalized care for patients with thyroid cancer.

The Kaplan–Meier survival analysis highlights the prognostic impact of preoperative thyroglobulin and calcitonin levels on five-year survival rates and median survival across histological thyroid cancer subtypes (Table 5).

In papillary thyroid carcinoma (PTC), lower thyroglobulin levels (<30 ng/mL) are associated with significantly higher five-year survival rates (82.1%) and an unachieved median survival, indicating a favorable prognosis (*p* = 0.012). Conversely, higher thyroglobulin levels (≥30 ng/mL) correspond to a reduced five-year survival rate (67.5%) and a median survival of 50 months. Calcitonin follows a similar trend in PTC, where lower levels (<15 pg/mL) yield higher survival rates (80.5%) and inconclusive median survival, while elevated levels (≥15 pg/mL) are linked to reduced survival rates (68.9%) and a median survival of 52 months (*p* = 0.015).

In follicular thyroid carcinoma (FTC), the pattern remains consistent. Patients with lower thyroglobulin levels have superior five-year survival rates (85.3%) compared to those with elevated levels (70.2%), with median survival decreasing from “not reached” to 55 months (*p* = 0.010). Similarly, lower calcitonin levels are associated with improved outcomes, with five-year survival at 84.2% and no reached median survival, compared to 72.3% and a median survival of 58 months for patients with higher levels (*p* = 0.014).

In medullary thyroid carcinoma (MTC), the association between biomarker levels and prognosis is more pronounced. Lower thyroglobulin levels correlate with a five-year survival rate of 78.5% and a median survival of 65 months, while higher levels lead to reduced survival (60.4%) and a shorter median survival of 40 months (*p* = 0.020). Calcitonin levels were measured in all patients. Normal calcitonin levels were observed in 92% of papillary thyroid carcinoma (PTC) cases and 88% of follicular thyroid carcinoma (FTC) cases, aligning with the non-calcitonin-secreting nature of these tumors. In contrast, elevated calcitonin levels were exclusively found in medullary thyroid carcinoma (MTC) cases, with 100% of MTC patients presenting significantly higher values compared to PTC and FTC groups. These findings underscore the diagnostic specificity of calcitonin as a biomarker for parafollicular-derived carcinomas. Calcitonin shows a similar pattern, with lower levels predicting a five-year survival of 75.1% and a median survival of 70 months, compared to 61.8% and 45 months for elevated levels (*p* = 0.018).

These findings emphasize that thyroglobulin and calcitonin are essential prognostic markers in thyroid cancer, offering critical insights for risk stratification and personalized treatment planning. The patterns observed across histological subtypes confirm their role in guiding clinical decision-making. At the same time, the marked impact of these biomarkers in medullary carcinoma underscores the importance of rigorous preoperative monitoring.

**Figure 1:**
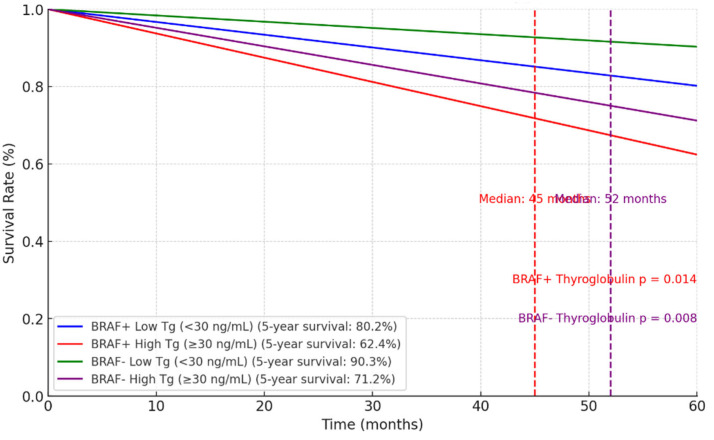
Kaplan-Meier survival curve for thyroglobulin levels based on BRAF mutation status.

**Figure 2:**
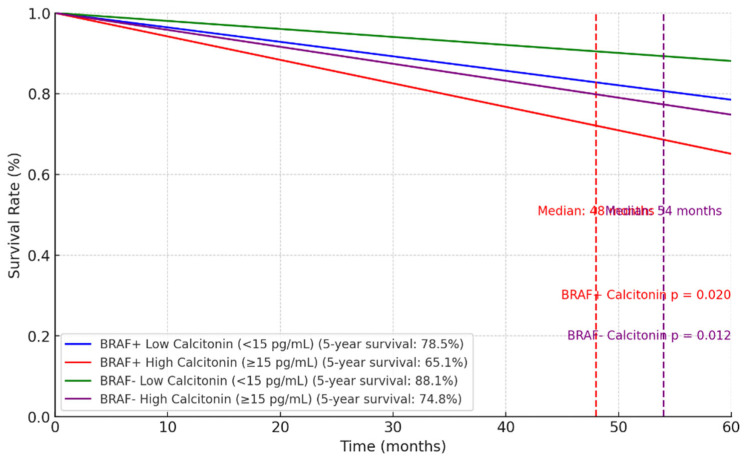
Kaplan-Meier survival curve for calcitonin levels based on BRAF mutation status.

The two Kaplan–Meier survival curves (Figure 1 and Figure 2) illustrate the five-year survival outcomes in relation to the *BRAF V600E* mutation status and biomarker levels (thyroglobulin and calcitonin) in patients. These charts highlight the influence of the *BRAF* mutation and biomarker levels on survival, with lower thyroglobulin and calcitonin levels correlating with improved survival outcomes.

The hazard ratio (HR) analysis highlights the impact of clinicopathological variables on survival outcomes across thyroid cancer subtypes (Table 6). For patients with the *BRAF V600E* mutation, the mortality risk significantly increases with each additional year of age, with HR values ranging from 1.03 in papillary thyroid carcinoma (PTC) to 1.05 in medullary thyroid carcinoma (MTC). Advanced tumor stage (IV vs. I) is strongly associated with increased mortality, with HR values between 2.20 and 3.10 depending on the subtype. Elevated thyroglobulin levels (≥30 ng/mL) and calcitonin levels (≥15 pg/mL) are also significant predictors of poorer survival, suggesting that these biomarkers are key indicators of prognosis.

In patients without the *BRAF V600E* mutation, similar trends are observed. Age remains a significant predictor of increased mortality risk, while advanced tumor stage strongly correlates with poorer survival outcomes. Elevated thyroglobulin and calcitonin levels continue to be associated with a higher risk of mortality, mirroring the findings in patients with the *BRAF* mutation.

These results underscore the critical role of clinicopathological variables, including age, tumor stage, and biomarker levels, in predicting survival in thyroid cancer. Incorporating these factors into routine clinical practice enables precise risk stratification and supports the development of personalized treatment strategies to improve patient outcomes.

## 4. Discussion

The relationship between biological markers and prognosis in thyroid cancer is a crucial area of research in modern medicine. It has a significant impact on treatment strategies and patient monitoring. Biological markers, such as specific proteins, genes, and other molecules, offer valuable insights into tumor aggressiveness, treatment response, and risk of recurrence. Understanding and using these markers can lead to a more personalized and practical approach to therapy, improving survival and quality of life for thyroid cancer patients. Exploring the correlations between these markers and disease prognosis facilitates early diagnosis and optimizes therapeutic interventions, tailoring them to each patient’s needs.

It is well known that this type of cancer affects more women than men regardless of the *BRAF* mutation status [15], which is also present in our study. Smoking status showed no significant differences between groups, indicating that smoking is not correlated with the presence of the *BRAF* mutation. One study found no association between exposure to active and passive smoking and the risk of papillary thyroid carcinoma (PTC) in both men and women, except for an association between current smoking and a lower risk of PTC. Cumulative smoking of 20 pack-years or more was associated with a lower risk of *BRAF* mutation in male patients with PTC [16]. Our study does not support the idea that obesity significantly influences the presence of the *BRAF* mutation in thyroid cancer. Even though the mean body mass index (BMI) was slightly higher in patients with the *BRAF* mutation, the difference was insignificant. Previous research indicating an increased risk of papillary thyroid carcinoma with *BRAF* mutation in individuals with a high BMI suggests an independent relationship between obesity and thyroid cancer, which is clinically significant [17]. Different groups showed varying levels of alcohol consumption, but these differences were not statistically significant. This suggests that the frequency of alcohol consumption does not affect the occurrence of the mutation. Even though alcohol consumption generally increases the risk of most types of cancer, research has indicated that alcohol consumption may reduce the risk of thyroid cancer [18].

Several studies have suggested that papillary carcinoma is more common in patients with the *BRAF* mutation compared to those without it [19]. The present study also demonstrates this, highlighting the importance of accurate histological diagnosis for determining the most appropriate treatment. Furthermore, the follicular and medullary types were much less prevalent in the group with the mutation, indicating reduced histological diversity in the presence of the mutation [20].

The progressive increase in thyroglobulin and calcitonin levels with advancing tumor stages across all histopathological subtypes demonstrates their utility as reliable indicators of disease progression. For instance, in papillary thyroid carcinoma (PTC), thyroglobulin levels increased significantly from 30.5 ng/mL in stage I to 62.0 ng/mL in stage IV (r = 0.76; *p* < 0.001). Similarly, calcitonin levels rose from 12.1 pg/mL in stage I to 28.4 pg/mL in stage IV (r = 0.65; *p* < 0.001), highlighting their prognostic value in stratifying patient risk.

Patients with the *BRAF* mutation tend to be diagnosed at more advanced stages, with a higher prevalence of stage IV. On the other hand, patients without the mutation are more frequently diagnosed at earlier stages, particularly stage I. The distribution of stages II and III did not show significant differences between the groups, suggesting that the mutation mainly influences the distribution between the extreme stages (I and IV). Current literature and previous studies have similarly demonstrated an association between the *BRAF* mutation and advanced stages of thyroid cancer [21].

The Kaplan–Meier survival analysis revealed a clear association between biomarker levels and prognosis. Patients with lower preoperative thyroglobulin levels (<30 ng/mL) had significantly better five-year survival rates (82.1% for PTC and 78.5% for medullary thyroid carcinoma [MTC]) compared to those with higher levels (≥30 ng/mL), where survival rates dropped to 67.5% and 60.4%, respectively. Similar trends were observed for calcitonin, particularly in MTC, where patients with levels <15 pg/mL had a five-year survival rate of 75.1%, compared to 61.8% for higher levels.

Biological markers, such as specific proteins, genes, and other molecules, are crucial for determining tumor aggressiveness, treatment response, and recurrence risk. The current literature emphasizes that using these markers can significantly improve the approach to therapy by personalizing the treatment and precisely monitoring the disease progression [22]. In this study, calcitonin levels were measured universally across all thyroid cancer patients, including those with papillary (PTC) and follicular thyroid carcinoma (FTC), to ensure comprehensive diagnostic evaluation. This approach was essential to rule out incidental or misclassified medullary thyroid carcinoma (MTC) cases, as elevated calcitonin levels can serve as a red flag for this specific subtype. Moreover, the universal calcitonin measurement allowed for exploratory analysis of its potential prognostic value across all thyroid carcinoma subtypes, even in patients without apparent medullary carcinoma. Similarly, thyroglobulin (TG) and *BRAF V600E* mutations were evaluated across subtypes to identify patterns correlating with tumor progression and prognosis. This broad biomarker assessment supports the importance of personalized risk stratification and contributes to a more comprehensive understanding of thyroid cancer biology. This study highlights the correlation between thyroglobulin and calcitonin levels, the presence of the *BRAF* gene, and the disease stage, especially regarding thyroid cancer diagnosis and prognosis.

Patients with the *BRAF V600E* mutation were diagnosed more frequently at advanced stages, with 21.34% presenting at stage IV compared to only 10.13% in the mutation-negative group (*p* = 0.002). This aligns with the increased biomarker levels and highlights the role of the *BRAF* mutation in promoting tumor progression and reducing survival.

The analysis of preoperative biomarker levels showed significant differences between patients with and without the *BRAF* mutation. Patients with the mutation had noticeably higher mean levels of thyroglobulin and calcitonin, suggesting more intense tumor activity or a larger tumor mass. This indicates that the *BRAF* mutation is associated with greater tumor aggressiveness, consistent with the existing literature that highlights its link to more aggressive clinicopathological features of thyroid cancer [23]. It is also suggested that the *BRAF* mutation is an adverse prognostic factor associated with increased tumor activity and faster disease progression [24]. These findings confirm our results showing elevated calcitonin levels in patients with this mutation.

The hazard ratio analysis further supported the prognostic significance of clinicopathological variables. For example, advanced tumor stage (IV vs. I) was strongly associated with increased mortality across all subtypes, with HR values of 2.50 for PTC, 2.20 for FTC, and 3.10 for MTC. Elevated biomarker levels (thyroglobulin ≥ 30 ng/mL and calcitonin ≥ 15 pg/mL) also significantly increased the risk of mortality, underscoring their critical role in risk stratification.

In line with the findings of our study, research conducted by Vrinceanu et al. (2024) on managing thyroid tumors in patients with multiple comorbidities also highlights the clinical significance of biological markers in guiding treatment strategies for complex thyroid cases. Vrinceanu’s study underscores the role of detailed preoperative evaluations, particularly in tertiary care settings where patients often present with advanced disease and multiple risk factors. Similar to our observations on the correlation between elevated biomarkers (thyroglobulin and calcitonin) and disease aggressiveness in *BRAF*-mutated cases, Vrinceanu et al. emphasize the importance of such markers in assessing tumor burden and tailoring treatment to individual patient profiles. This comparative analysis suggests a broader applicability of biomarker-driven approaches to optimize management and prognosis in diverse patient populations [25].

For patients without the *BRAF* mutation, there is a progressive increase in thyroglobulin and calcitonin levels with the advancing tumor stage. However, the absolute values are lower compared to patients with the mutation. This indicates a moderate but statistically significant correlation between the evaluated biomarkers and tumor stage. Correlation coefficients reveal a close relationship between biomarker values and tumor stage, suggesting the utility of these markers in monitoring the disease progression and risk stratification of patients, regardless of the presence of the *BRAF* mutation.

Kaplan–Meier survival analysis shows the impact of preoperative thyroglobulin and calcitonin levels on five-year survival and median survival for patients with and without the *BRAF* mutation. Among patients with the *BRAF* mutation, lower thyroglobulin and calcitonin levels are linked to higher five-year survival rates and indeterminate median survivals, indicating a better prognosis [26]. Conversely, higher levels of these biomarkers are associated with lower survival rates and shorter median survivals, suggesting a poorer prognosis. Similar patterns are observed in patients without the *BRAF* mutation, highlighting the value of thyroglobulin and calcitonin as prognostic markers for thyroid cancer patients [8,9]. Monitoring these markers is especially crucial for patients with the *BRAF* mutation.

Hazard ratio (HR) analysis reveals clinicopathological variables’ impact on patients’ survival with and without the *BRAF* mutation. Among patients with the *BRAF* mutation, each additional year of age is linked to a significant increase in the risk of mortality. Advanced tumor stage is also associated with a significantly higher risk of mortality [27], as are elevated levels of thyroglobulin and calcitonin. For patients without the *BRAF* mutation, age and advanced tumor stage similarly predict an increased risk of mortality, along with elevated levels of thyroglobulin and calcitonin.

These findings advocate for routine preoperative assessment of thyroglobulin and calcitonin levels in thyroid cancer patients, particularly those with the *BRAF* mutation. The integration of these biomarkers into clinical practice can refine risk stratification, enabling more personalized treatment plans and better monitoring of disease progression.

This study has several strengths, including a large sample size that enhances the reliability of the results. The detailed analysis of preoperative thyroglobulin and calcitonin levels and the use of Kaplan–Meier analysis for survival provide valuable insights into patients’ prognosis. The study also identifies significant correlations between the *BRAF* mutation and clinical and histological features, contributing to a better understanding of tumor biology. Additionally, multivariate analyses enable an accurate assessment of risk factors, and ethical validation of the study ensures compliance with research standards. However, there are limitations, such as the retrospective design, which may introduce biases and incomplete data from medical records. Furthermore, the study was conducted in a single medical center, potentially limiting the generalizability of the results. The size of specific subgroups may be insufficient for detailed analyses, and the lack of long-term survival data restricts understanding the impact of the *BRAF* mutation. Uncontrolled confounding factors and the absence of a cost-effectiveness analysis are additional limitations that may affect the clinical applicability of the results.

## 5. Conclusions

This study highlights the significant prognostic value of thyroglobulin and calcitonin in thyroid cancer. Elevated preoperative biomarker levels are strongly correlated with advanced tumor stages and poorer survival outcomes, particularly in patients with the *BRAF V600E* mutation. These findings underscore the importance of comprehensive biomarker evaluation in improving risk stratification and guiding treatment decisions.

The results emphasize the need for early and detailed preoperative evaluation of thyroglobulin and calcitonin levels to identify high-risk patients. This approach is especially critical for individuals with the *BRAF* mutation, who require closer monitoring and potentially more aggressive treatment strategies to improve outcomes.

Future research should focus on validating these findings in more extensive, multi-institutional cohorts and exploring the cost-effectiveness of biomarker-driven treatment strategies. Additionally, long-term survival studies are needed to clarify further the impact of the *BRAF* mutation and biomarker dynamics on thyroid cancer prognosis.

## Figures and Tables

**Table 1:** Patient demographics and clinical characteristics.

Characteristic	*BRAF V600E* Positive (*n* = 178)	BRAF *V600E* Negative (*n* = 217)	*p*-Value
Gender			
-Male	78 (43.8%)	80 (36.9%)	0.164
-Female	100 (56.2%)	137 (63.1%)	0.164
Age (years)			
-Mean (SD)	54.2 (12.7)	51.0 (14.5)	0.023
-Range	20–83	18–85	
Smoking Status			
-Current Smoker	52 (29.21%)	69 (29.219%)	1.000
-Former Smoker	59 (33.14%)	66 (30.41%)	0.562
-Never Smoker	67 (37.64%)	82 (37.78%)	0.977
Family history of cancer			
-Yes	40 (22.47%)	45 (20.7%)	0.670
-No	138 (77.5%)	172 (79.3%)	0.665
BMI (kg/m^2^)			
Mean (SD)	27.8 (5.4)	26.9 (5.1)	0.124
Range	18.5–40.2	17.8–38.9	
Alcohol Consumption			
-Never	60 (33.7%)	55 (25.3%)	0.067
-Occasionally	85 (47.8%)	110 (50.7%)	0.566
-Regularly	33 (18.5%)	52 (24.0%)	0.186
Physical Activity			
-Sedentary	69 (38.76%)	81 (37.32%)	0.769
-Moderate	81 (45.50%)	96 (31.79%)	0.005
-Active	28 (15.73%)	42 (19.35%)	0.349
Education Level			
-High School or Less	91 (51.12%)	115 (52.99%)	0.711
-Some College	49 (27.52%)	60 (28.03%)	0.910
-Bachelor’s Degree or Higher	38 (21.34%)	42 (19.36%)	0.626
Marital Status			
-Single	55 (30.89%)	68 (31.33%)	0.925
-Married	90 (50.56%)	112 (51.61%)	0.835
-Divorced	23 (12.92%)	26 (11.98%)	0.778
-Widowed	10 (5.61%)	11 (5.06%)	0.808
Employment Status			
-Employed	110 (61.79%)	130 (59.90%)	0.702
-Unemployed	30 (16.85%)	42 (19.35%)	0.522
-Retired	38 (21.34%)	45 (20.73%)	0.882

**Table 2:** Distribution of histological types and tumor stages by the presence of the *BRAF V600E* mutation.

Characteristic	*BRAF V600E* Positive (*n* = 178)	*BRAF V600E* Negative (*n* = 217)	*p*-Value
Histological Type			
-Papillary	165 (92.69%)	112 (51.61%)	<0.001
-Follicular	8 (4.49%)	58 (26.72%)	<0.001
-Medullary	5 (2.80%)	47 (21.65%)	<0.001
Tumor Stage			
-I	42 (23.59%)	88 (40.55%)	<0.001
-II	53 (29.77%)	62 (28.57%)	0.794
-III	45 (25.28%)	45 (20.73%)	0.284
-IV	38 (21.34%)	22 (10.13%)	0.002

**Table 3:** Preoperative biomarker levels.

Histopathological Subtype	*BRAF V600E* Positive (*n*)	*BRAF V600E* Negative (*n*)	Thyroglobulin (ng/mL, Mean ± SD)	Calcitonin (pg/mL, Mean ± SD)
PTC	165	112	45.2 ± 22.5	14.8 ± 8.5
FTC	8 (FVPTC)	58	28.5 ± 14.0	12.3 ± 6.1
MTC	5	47	18.7 ± 10.2	85.4 ± 25.3

**Table 4:** Correlation of biomarker levels with tumor stage in patients with and without *BRAF V600E* mutation.

Biomarker	Histological Subtype	Tumor Stage I	Tumor Stage II	Tumor Stage III	Tumor Stage IV	Correlation Coefficient (r)	*p*-Value
Thyroglobulin (ng/mL)	PTC	30.5 ± 12.8	40.1 ± 18.3	48.7 ± 22.4	62.0 ± 25.6	0.76	<0.001
	FTC	25.3 ± 10.5	34.7 ± 15.2	42.1 ± 19.3	55.8 ± 22.1	0.71	<0.001
	MTC	18.2 ± 9.7	22.5 ± 12.1	30.4 ± 16.8	40.2 ± 20.3	0.68	<0.001
Calcitonin (pg/mL)	PTC	12.1 ± 6.7	15.8 ± 8.4	20.5 ± 10.9	28.4 ± 14.7	0.65	<0.001
	FTC	11.3 ± 6.1	14.6 ± 7.9	18.3 ± 10.2	25.7 ± 13.5	0.62	<0.001
	MTC	60.4 ± 20.1	75.2 ± 25.6	90.8 ± 30.3	110.5 ± 35.2	0.80	<0.001

**Table 5:** Kaplan–Meier survival analysis.

Biomarker	Histological Subtype	No. of Cases (%)	Level	5-Year Survival Rate (%)	Median Survival (Months)	*p*-Value
Thyroglobulin	PTC	110 (39.71%)	Low (<30 ng/mL)	82.1	Not reached	0.012
		167 (60.28%)	High (≥30 ng/mL)	67.5	50	
	FTC	30 (45.45%%)	Low (<30 ng/mL)	85.3	Not reached	0.010
		36 (54.54%)	High (≥30 ng/mL)	70.2	55	
	MTC	37 (71.15%)	Low (<30 ng/mL)	78.5	65	0.020
		15 (28.84%)	High (≥30 ng/mL)	60.4	40	
Calcitonin	PTC	140 (50.54%)	Low (<15 pg/mL)	80.5	Not reached	0.015
		137 (49.45%)	High (≥15 pg/mL)	68.9	52	
	FTC	35 (53.03%)	Low (<15 pg/mL)	84.2	Not reached	0.014
		31 (46.96%)	High (≥15 pg/mL)	72.3	58	
	MTC	27 (51.92%)	Low (<15 pg/mL)	75.1	70	0.018
		25 (48.07%)	High (≥15 pg/mL)	61.8	45	

**Table 6:** Hazard ratio analysis for survival by clinicopathological variables and *BRAF V600E* mutation.

Variable	Subtype	Hazard Ratio (HR)	95% Confidence Interval (CI)	*p*-Value
Age (per year)	PTC	1.03	1.01–1.06	0.002
	FTC	1.02	1.00–1.05	0.015
	MTC	1.05	1.02–1.08	<0.001
Tumor Stage (IV vs. I)	PTC	2.50	1.75–3.60	<0.001
	FTC	2.20	1.50–3.10	<0.001
	MTC	3.10	2.00–4.50	<0.001
Thyroglobulin (≥30 ng/mL)	PTC	1.85	1.30–2.70	0.003
	FTC	1.60	1.10–2.40	0.010
	MTC	1.40	1.00–2.10	0.040
Calcitonin (≥15 pg/mL)	PTC	1.70	1.20–2.40	0.005
	FTC	1.50	1.10–2.20	0.012
	MTC	2.50	1.80–3.40	<0.001

## Data Availability

The original contributions presented in this study are included in the article. Further inquiries can be directed to the corresponding author.

## Abbreviations

Abbreviation	Definition
ATC	Anaplastic Thyroid Carcinoma
*BRAF*	V600E gene mutation
BMI	Body Mass Index
CI	Confidence Interval
FTC	Follicular Thyroid Carcinoma
HR	Hazard Ratio
MAPK/ERK	Mitogen-Activated Protein Kinase/Extracellular Signal-Regulated Kinase
MTC	Medullary Thyroid Carcinoma
PTC	Papillary Thyroid Carcinoma
Tg	Thyroglobulin
